# Central Gαi_2_ Protein Mediated Neuro-Hormonal Control of Blood Pressure and Salt Sensitivity

**DOI:** 10.3389/fendo.2022.895466

**Published:** 2022-06-28

**Authors:** Razie Amraei, Jesse D. Moreira, Richard D. Wainford

**Affiliations:** ^1^Department of Pathology and Laboratory Medicine, Boston University School of Medicine, Boston, MA, United States; ^2^Whitaker Cardiovascular Institute, Boston University School of Medicine, Boston, MA, United States; ^3^Department of Medicine, Boston University School of Medicine, Boston, MA, United States; ^4^Department of Pharmacology & Experimental Therapeutics, Boston University School of Medicine, Boston, MA, United States

**Keywords:** Gαi_2_ proteins, paraventricular nucleus, hypertension, renal nerves, salt sensitivity

## Abstract

Hypertension, a major public health issue, is estimated to contribute to 10% of all deaths worldwide. Further, the salt sensitivity of blood pressure is a critical risk factor for the development of hypertension. The hypothalamic paraventricular nucleus (PVN) coordinates neuro-hormonal responses to alterations in plasma sodium and osmolality and multiple G Protein-Coupled Receptors (GPCRs) are involved in fluid and electrolyte homeostasis. In acute animal studies, our laboratory has shown that central Gαi/o subunit protein signal transduction mediates hypotensive and bradycardic responses and that Gz/q, proteins mediate the release of arginine vasopressin (AVP) and subsequent aquaretic responses to acute pharmacological stimuli. Extending these studies, our laboratory has shown that central Gαi_2_ proteins selectively mediate the hypotensive, sympathoinhibitory and natriuretic responses to acute pharmacological activation of GPCRs and in response to acute physiological challenges to fluid and electrolyte balance. In addition, following chronically elevated dietary sodium intake, salt resistant rats demonstrate site-specific and subunit-specific upregulation of Gαi_2_ proteins in the PVN, resulting in sympathoinhibition and normotension. In contrast, chronic dietary sodium intake in salt sensitive animals, which fail to upregulate PVN Gαi_2_ proteins, results in the absence of dietary sodium-evoked sympathoinhibition and salt sensitive hypertension. Using *in situ* hybridization, we observed that Gαi_2_ expressing neurons in parvocellular division of the PVN strongly (85%) colocalize with GABAergic neurons. Our data suggest that central Gαi_2_ protein-dependent responses to an acute isotonic volume expansion (VE) and elevated dietary sodium intake are mediated by the peripheral sensory afferent renal nerves and do not depend on the anteroventral third ventricle (AV3V) sodium sensitive region or the actions of central angiotensin II type 1 receptors. Our translational human genomic studies have identified three G protein subunit alpha I2 (GNAI2) single nucleotide polymorphisms (SNPs) as potential biomarkers in individuals with salt sensitivity and essential hypertension. Collectively, PVN Gαi_2_ proteins-gated pathways appear to be highly conserved in salt resistance to counter the effects of acute and chronic challenges to fluid and electrolyte homeostasis on blood pressure *via* a renal sympathetic nerve-dependent mechanism.

## Introduction

Hypertension is a critical public health issue that affects approximately 1 in 2 U.S. adults (1). It is the leading risk factor for chronic kidney disease, myocardial infarction, and stroke, and is estimated to result in approximately 10% of all global deaths (1). Accumulating evidence shows that excess dietary salt intake increases the risk for both hypertension and adverse cardiovascular outcomes (2, 3). Despite the current approaches to sodium reduction, approximately 90% of United States adults exceed the American Heart Association recommended daily intake of sodium (<3200mg) (4). The excess intake of dietary salt increases cardiovascular risk due to the salt sensitivity of blood pressure, which is defined as an exaggerated pressor response to elevated dietary sodium intake (5–7), that increases the risk of hypertension. Significantly, the prevalence of the salt sensitivity of blood pressure is estimated to be present in 25% of normotensive to 50% of hypertensive individuals (8) and represents a major public health issue.

Several studies have demonstrated that excess sympathetic nervous system activity contributes to both the development and maintenance of hypertension (5, 9–15). Multiple animal models, including angiotensin II infused rats (16), spontaneously hypertensive rats (17), DOCA-salt treated rats (18), and mouse models (19, 20) have provided mechanistic insight into the role of G-Protein Coupled Receptors (GPCRs) proteins across the cardiovascular and nervous systems (21, 22). It is well established that multiple GPCRs influence sympathetic nervous system activity, fluid and electrolyte homeostasis and blood pressure regulation. The focus of this review is predominantly on recent *in vivo* studies from our laboratory that investigate the impact of brain GPCR Gα-subunit protein gated signaling in the regulation of fluid and electrolyte balance, sympathetic outflow and the regulation of blood pressure in response to acute and chronic challenges to sodium balance and how this influences the salt sensitivity of blood pressure.

## G-Protein Coupled Receptors and Gαi-subunit Proteins

**Figure 1:**
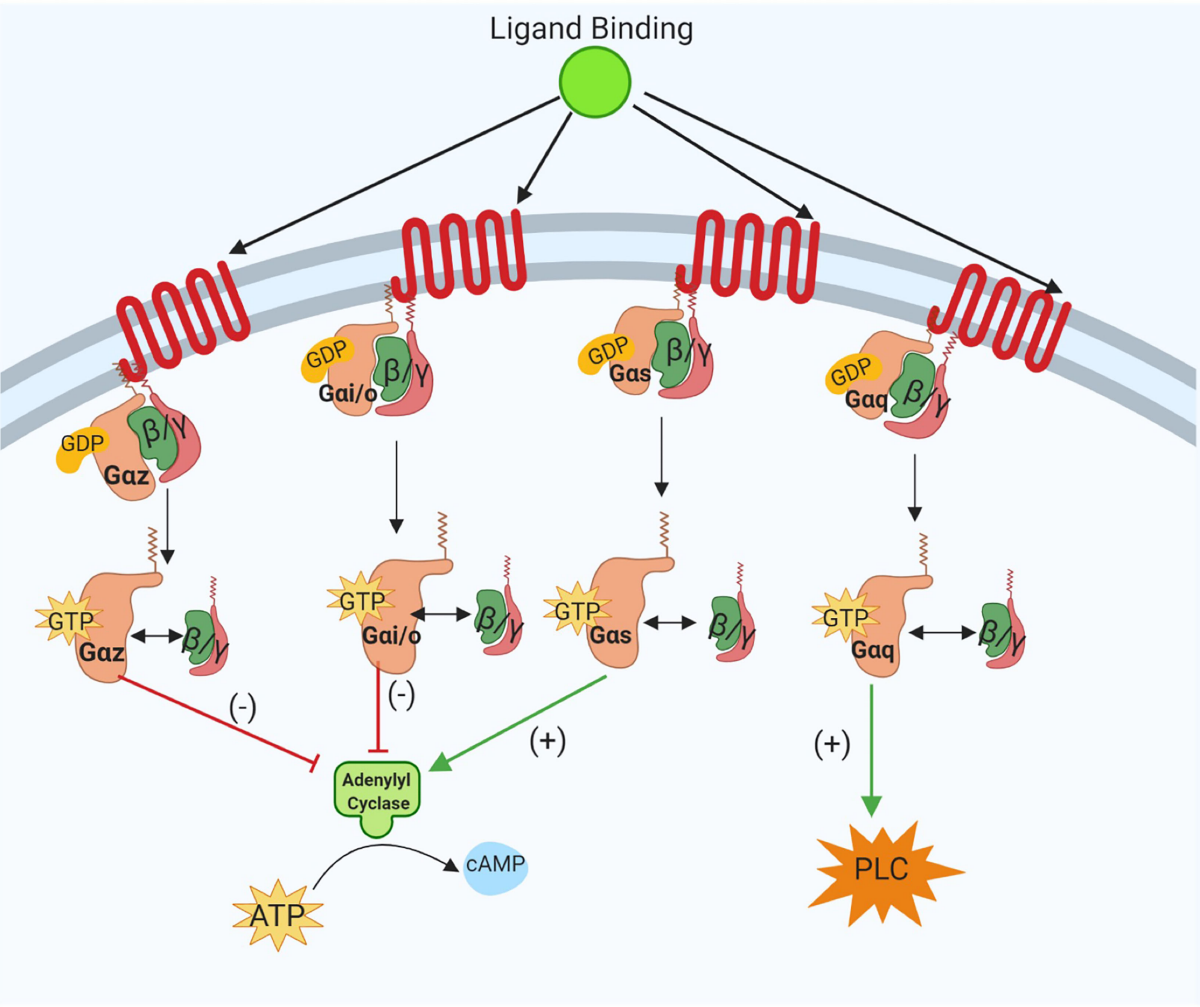
Schematic representation of the canonical intracellular Gα subunit signal transduction pathways activated following ligand binding at G-protein Coupled Receptors (GPCRs). The specificity of Gαi/o, Gαs, Gαz, and Gαq subunit activation in downstream signaling pathways is shown. As illustrated dissociation of Gαz and Gαi/o subunits reduces cyclic AMP (cAMP) and GTP-bound Gαs increases intracellular cAMP levels. Activation of Gαq subunits evokes increased phospholipase C (PLC) signaling activity. Image was generated using BioRender.

G-protein coupled receptors are 7-transmembrane receptors which have an extracellular binding domain and intracellular protein interactions with heterotrimeric G proteins consisting of an α-subunit and β/γ-dimer (23). In the absence of ligand binding, all G protein subunits are associated with the receptor, and the α-subunit is bound to guanine diphosphate (GDP). Upon activation, the α-subunit will exchange GDP for guanine triphosphate (GTP), followed by dissociation of α-subunit and β/γ-dimer from the receptor to initiate signal transduction. The function of the α-subunit is dependent upon its sub-classification and downstream effector molecules. The main four classes of α-subunits are Gαi/o, Gαs, Gαz, and Gαq. Principally, Gαi/o and Gαz subunits inhibit the activity of adenylyl cyclase, thus reducing intracellular levels of cyclic adenosine monophosphate (cAMP) and the subclass of Gαs proteins enhance adenylyl cyclase activity which leads to increased cAMP levels. Lastly, Gαq proteins activate phospholipase C (PLC), promote production of intracellular inositol triphosphate (IP3) and regulate intracellular calcium release (23) (**Figure 1**). Gα-subunit selectivity is critical for subsequent intracellular signal transduction and *in vitro* and *in vivo* models of GPCRs signaling have demonstrated the specificity of each subclass of Gα proteins (24).

## GPCR Signaling and Blood Pressure Regulation

GPCR signaling pathways impact multiple aspects of cardiovascular system and a wide variety of GPCRs are responsible for the regulation of blood pressure. As GPCRs are expressed in various cell types in the brain, heart, blood vessels, kidney etc. (25) their signaling activity can alter heart rate, vascular resistance and/or blood volume. The main receptor systems involved in cardiovascular regulation, including the α and β adrenoceptors, muscarinic and cholinergic receptors are GPCRs (26, 27). Similarly, multiple endogenous ligands that influence blood pressure such as norepinephrine (28), angiotensin II (29), acetylcholine (30), endothelin (31) etc. are ligands for GPCRs. The expression and function of GPCRs is tightly regulated by different mechanisms. For example, G-Protein coupled receptor kinases (GRKs) can modulate adrenergic receptor responses and elevations in GRK2 and GRK5 in vascular smooth muscle cells and lymphocytes are associated with human hypertension (32). Mediators of GPCR signaling represent potential new targets for blood pressure control and this review will focus on recent advances in our understanding of the actions of central Gα-subunit proteins in the neurohumoral control of blood pressure.

## Functional Selectivity of Central Gαi-subunit Protein-Gated Mediated Cardio-Renal Signal Transduction

The central mechanisms regulating short and long-term cardiovascular homeostasis and renal excretory function involve multiple GPCR systems (e.g., the α_2_ adrenoceptor). Our initial *in vivo* studies in conscious Sprague Dawley rats demonstrated that in response to direct pharmacological stimulation of a brain GPCR downstream activation of selective Gα-subunit signaling mediates cardiovascular vs. renal excretory responses (33). Utilizing acute central administration of Nociceptin/Orphanin FQ (N/OFQ), the agonist of the Nociceptin/Orphanin FQ (NOP) GPCR, which signals *via* Gαi/o, Gαz and Gαq proteins to evoke hypotension, bradycardia and diuresis (34), we observed a differential effect of central inhibition of Gαi/o vs. downregulation of Gαz/Gαq proteins on cardiovascular vs. renal function. Following inhibition of the activity of central Gαi/o proteins with pertussis toxin we observed abolishment of centrally administered N/OFQ bradycardia and hypotension with no effect on the diuretic effect evoked by N/OFQ (33). In contrast selective individual targeted oligodeoxynucleotide (ODN)-mediated downregulation of central Gαz/Gαq proteins, which reduced target protein expression ~85%, markedly blunted (Gαz) or augmented (Gαq) the diuretic response to central N/OFQ without impacting the cardiovascular depressor effects of N/OFQ (33). These studies provided the first *in vivo* evidence in conscious animals of the functional selectively of Gα-subunit signaling in response to direct pharmacological GPCR activation to influence cardiovascular vs. renal excretory function.

### Central Gαz/Gαq-Subunit Regulation of Arginine Vasopressin Secretion

To assess the potential mechanism underlying the impact of the modulation of the expression of central Gαz/Gαq subunit proteins on diuresis in response to N/OFQ we assessed plasma AVP levels in response to central administration of N/OFQ. It is well established that N/OFQ evokes aquaresis, in part, *via* the suppression of plasma AVP release. In water-restricted Sprague Dawley rats the ability of central N/OFQ to reduce plasma AVP levels was differently modulated by selective ODN-mediated downregulation of central Gαz/Gαq proteins with central Gαz down regulation blunting AVP suppression and central Gαq downregulation augmenting N/OFQ-mediated AVP suppression (33). Given the central role of AVP in fluid homeostasis and blood pressure regulation we extended these studies to assess the potential role of central brain Gαz/Gαq proteins in the regulation of blood pressure, fluid homeostasis and vasopressin secretion in the Dahl rat model of salt sensitive hypertension. Following 21-days of high dietary salt intake the Dahl salt sensitive (DSS), but not the salt resistant Dahl salt resistant (DSR) rat, exhibited salt sensitive hypertension, elevated plasma AVP levels and positive water balance (35). Given our prior finding of an influence of central Gαz/Gαq proteins on AVP release we assessed the influence of high dietary salt intake on central Gαz/Gαq protein expression in DSS and DSR rats. Our data show that a chronic high salt intake evoked selective endogenous down-regulation of Gαq, but not Gαz, proteins in the hypothalamic paraventricular nucleus (PVN) in DSR, but not DSS rats. In high dietary salt-challenged DSS rats acute selective targeted ODN-mediated down-regulation of central Gαq proteins returned plasma vasopressin to control levels, decreased dietary salt-induced water retention and restored the aquaretic response to N/OFQ to that seen in normotensive normal salt maintained DSS rats (35). These data provide the first evidence of the neurohumoral control of AVP secretion and subsequent aquaresis by central, likely PVN specific, Gαz/Gαq proteins and suggest that targeted down regulation of PVN Gαq proteins may represent an approach to prevent AVP hypersecretion in pathological states exhibiting AVP dysregulation.

### Central Gαi_2_ Proteins and Functional Selectivity of α_2_ Adrenoceptor Signal Transduction

To extend our initial findings that Gαi/o proteins mediate the cardiovascular depressor responses to central N/OFQ we elected to aim to identify which specific Gαi/o protein can selectively mediate the hypotensive and/or bradycardic responses to a classical anti-hypertensive agent, the α_2_ adrenoceptor agonist, Guanabenz. It is well established that following ligand binding to α_2_ adrenoceptor signal transduction can occur *via* downstream Gαi(1-3), Gα (o), Gα (s) subunit protein-gated pathways (36). To investigate the potential role(s) of individual central Gα subunit proteins we selectively down regulated individual brain Gαi_1_, Gαi_2_, Gαi_3_, Gαo, and Gαs subunit proteins by central pre-treatment with target specific oligodeoxynucleotide probes. In conscious rats, pre-treated with a control scrambled (SCR) ODN sequence that did not impact the expression of any tested brain Gα-subunit protein, central administration of Guanabenz, decreased mean arterial pressure (MAP) and heart rate (HR), and produced marked diuretic and natriuretic responses. In contrast, selective central Gαi_2_ protein down regulation blunted both the natriuretic and hypotensive responses to Guanabenz (36) with no impact on the bradycardic and diuretic response. Additionally, targeted Gαs down regulation converted the typical Guanabenz evoked hypotensive response into an immediate increase in MAP. Suggesting that multiple Gα-subunit proteins mediate the bradycardic response to guanabenz individual Gα-subunit protein targeting had no impact on the observed bradycardic response. Future studies, in which multiple Gα-subunit proteins are down regulated simultaneously, are required to identify the pathways mediating bradycardia. These studies suggested a central role of brain Gαi_2_ proteins in the regulation of blood pressure and the renal excretion of sodium.

### Central Gαi_2_ Proteins and Acute Natriuresis

An intravenous (i.v) isotonic volume expansion (VE) is a classical physiological challenge that evokes profound diuresis and natriuresis, independently from changes in blood pressure. In response to a 5% bodyweight an i.v. isotonic saline VE downregulation of brain Gαi_2_ subunits proteins abolished the suppression of renal sympathetic nerve activity and attenuated the natriuretic response compared to the profound suppression of RSNA and natriuretic response observed in control SCR ODN-pretreated rats (37). Additionally, bilateral renal denervation, which removes the influence of the renal sympathetic nerves on the kidneys, prevented the attenuation of the natriuretic response to i.v. VE following intracerebroventricular (ICV) ODN-mediated Gαi_2_ protein down regulation (37). Demonstrating the direct role of PVN Gαi_2_ proteins in the attenuated natriuretic response to an acute VE selective downregulation of PVN-specific Gαi_2_ proteins attenuated the natriuresis to an acute 5% body weight VE. To confirm that the PVN mediates Gαi_2_ protein-dependent natriuretic responses to an acute i.v. VE in a renal nerve dependent manner we also conducted studies in which the influence of the renal sympathetic nerves was removed by bilateral denervation (38). Our laboratory has recently reported that selective afferent renal nerve ablation prior to an acute i.v VE attenuates the natriuretic and PVN sympathoinhibitory response in Sprague Dawley rats (39). Given the sensory afferent renal nerves project to the spinal cord, and subsequent rostral projections may occur with the PVN, we speculate that the afferent renal nerves may modulate PVN Gαi_2_ protein signaling. Through the combination of selective afferent renal nerve ablation, alone or in combination with central Gαi_2_ protein down regulation we have established that brain Gαi_2_ protein-dependent responses to an acute i.v. VE involve activation of the sensory afferent renal nerves (40).

To validate our findings of a central role of Gαi_2_ proteins in the natriuretic responses to alterations in sodium homeostasis we examined the natriuretic response to an i.v. 1M NaCl infusion (20μl/min), that evokes profound natriuresis without altering blood pressure (41). In control SCR-ODN pretreated animals a 1M NaCl infusion produced natriuresis and robust decreases in both plasma norepinephrine and plasma renin activity (PRA). In contrast, in rats in which central Gαi_2_ proteins were down regulated, the natriuretic and sympathoinhibitory responses, but not the suppression of PRA, were attenuated (41). As observed in our acute VE studies bilateral renal denervation attenuated the blunted natriuretic and sympathoinhibitory responses to ODN-mediated downregulation of Gαi_2_ proteins during 1M NaCl loading. Further, in salt resistant Sprague Dawley rats in response to an acute i.v. bolus 3M NaCl (0.14ml/100g) hypertonic challenge, which raises blood pressure in addition to increasing plasma sodium, we observed attenuated natriuresis and a failure to return blood pressure to baseline levels following central Gαi_2_ protein down regulation (42). Collectively, these data highlight a newly discovered role of brain, and PVN specific, Gαi_2_ protein in the endogenous central sympathoinhibitory pathways, including the suppression of renal sympathetic nerve traffic, to mediate natriuresis in response to acute challenges to sodium homeostasis to maintain normotension.

## Gαi_2_ Proteins and the Salt Sensitivity of Blood Pressure

To examine the potential effect of dietary salt intake on endogenous central Gαi_2_ subunit protein expression, we employed 7-days dietary sodium restriction or excess in the salt resistant normotensive Sprague Dawley rat. Following 7-days of dietary sodium restriction PVN Gαi_2_ protein expression was markedly reduced. In contrast excess dietary sodium intake evoked a significant upregulation of Gαi_2_ subunit protein expression (37). The sodium-mediated alterations in Gαi_2_ protein levels were highly specific to the PVN and there was no impact of dietary sodium intake on the levels of Gαi_1_, Gαi_3_, or Gαo subunit proteins in any examined brain region (37). Significantly, in rats treated for 7-days high salt intake acute down-regulation of brain Gαi_2_ protein levels evoked sodium retention, global sympathoexcitation, and a significant elevation in blood pressure.

Extending this initial observation, we investigated the impact of central Gαi_2_ proteins on long-term blood pressure regulation during chronic elevations in dietary salt-intake. Replicating our prior study, we observed that 21-days high salt intake (8% NaCl) in normotensive salt resistant Sprague Dawley rats evoked a PVN-specific increase in Gαi_2_ protein levels that was accompanied by the suppression of plasma norepinephrine content and the cardiovascular depressor response to ganglionic blockade (markers of reduced sympathetic tone) (43). In contrast, in Gαi_2_ ODN pretreated animals, in which the expression of central Gαi_2_ proteins is ~85% reduced, a high salt intake resulted in the salt sensitivity of blood pressure which was associated with significant increases in plasma norepinephrine and vascular tone, suggestive of sympathoexcitation, and a rightward shift in the pressure-natriuresis curve. In Sprague Dawley rats in which the expression of central Gαi_2_ proteins is reduced by ICV ODN infusion bilateral renal denervation attenuated the observed increase in sympathetic outflow and prevented the development of the salt sensitivity of blood pressure (43). Validating these findings, we conducted subsequent studies involving ODN-mediated downregulation of Gαi_2_ in both DSR and Dahl sensitive (DSS) rats. As observed in the Sprague Dawley rat a 21-day high salt intake evoked PVN-specific upregulation of PVN Gαi_2_ proteins in the DSR phenotype. In contrast a high salt intake had no impact on the expression of Gαi_2_ proteins in the DSS rat. In DSR rats, Gαi_2_ ODN pretreatment prior to high salt intake evoked sympathetically mediated salt-sensitive hypertension, as determined by radiotelemetry and plasma norepinephrine levels (44). In these DSR rats there was an immediate rapid elevation in MAP of approximately 20 mmHg in a 3-day period followed by a gradual persistent increase in blood pressure. As observed in the Sprague Dawley rat the development of Gαi_2_ OD-mediated salt sensitivity of blood pressure was renal sympathetic nerve dependent (44). In DSS rats, which are an established model of salt sensitive hypertension, downregulation of Gαi_2_ proteins exacerbated the magnitude of salt sensitive hypertension. To investigate the impact of dietary sodium-evoked PVN Gαi_2_ protein upregulation on the development of salt sensitivity in the DSS we conducted studies using 8-congenic DSS rats, that contain chromosome 8 encoding the GNAI2 gene from the salt resistant Brown Norway rats (45). In response to high dietary sodium intake 8-congenic DSS rats exhibit increased PVN Gαi_2_ protein expression and attenuated salt sensitive hypertension, sodium retention, and sympathoexcitation compared to DSS rats (44).

**Figure 2:**
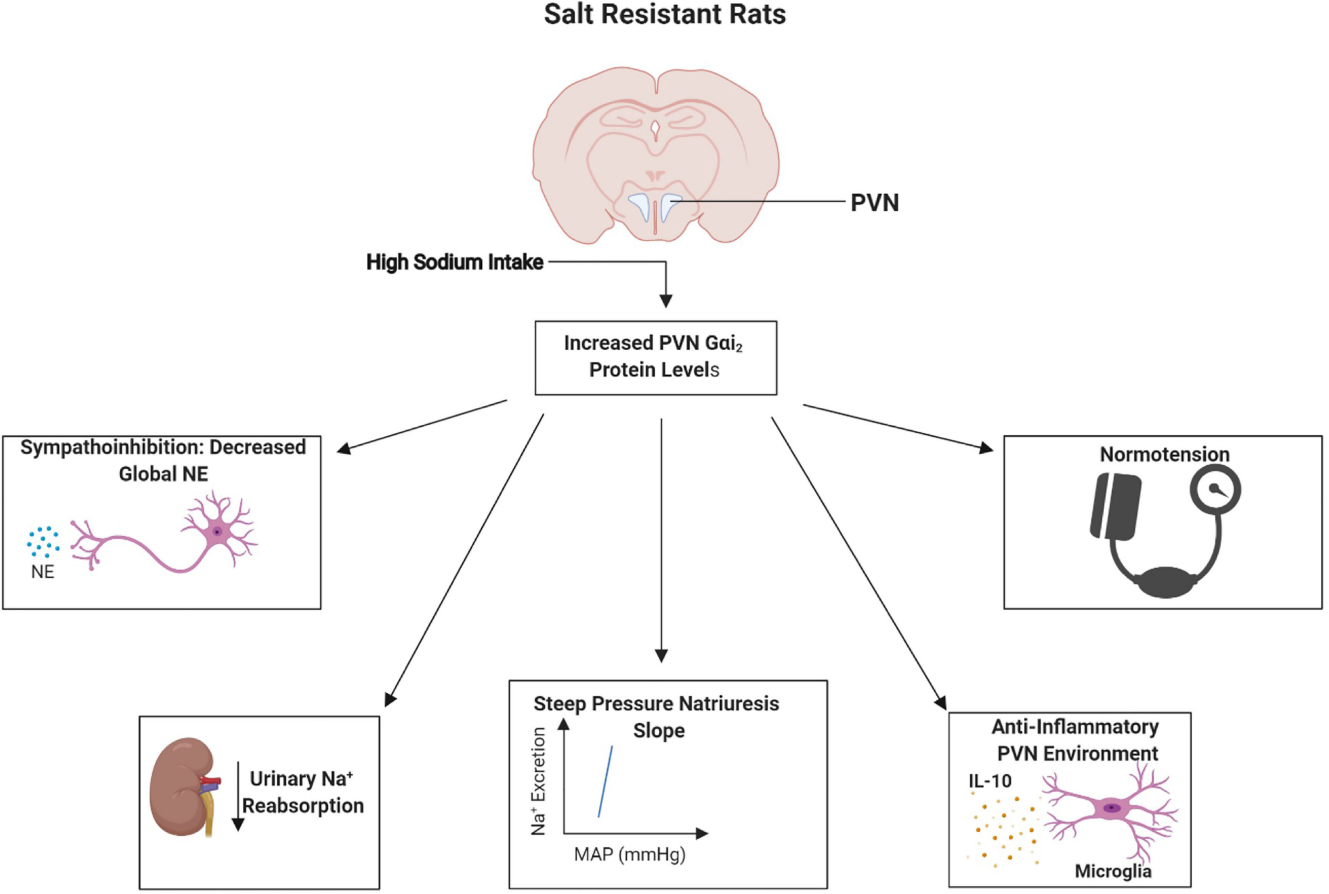
Schematic representation of the proposed physiological role of paraventricular nucleus (PVN) Gαi_2_ proteins in salt resistance. In response to high dietary-sodium intake, upregulation of PVN Gαi_2_ proteins acts as peripherally sensed sodium-responsive “anti-hypertensive” pathway that mediates systemic sympathoinhibition, natriuresis and central anti-inflammatory responses to maintain normotension and a salt resistant phenotype. Image was generated using BioRender.

**Figure 3:**
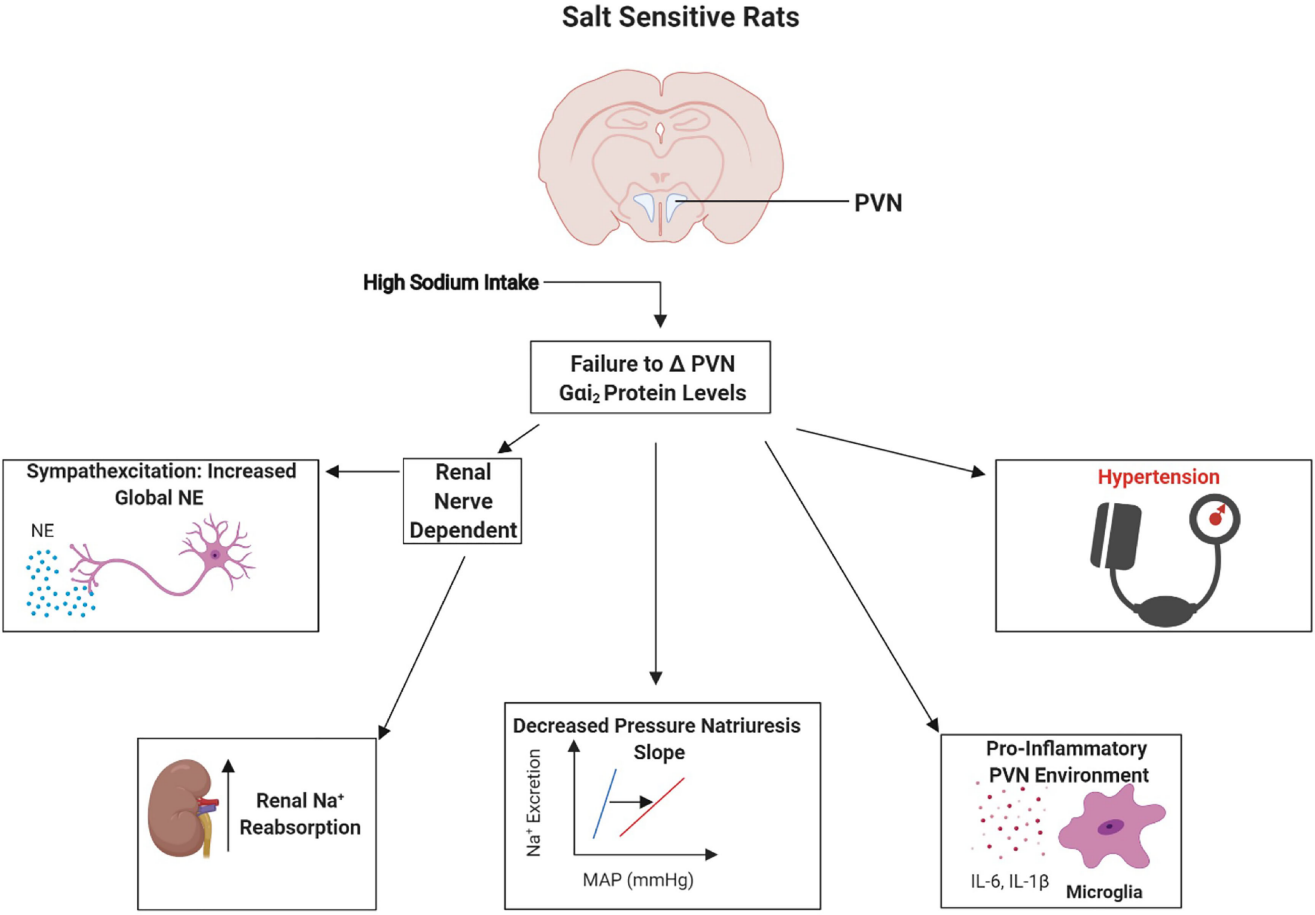
Schematic representation of the proposed physiological role of paraventricular nucleus (PVN) Gαi_2_ proteins in the development of the salt sensitivity of blood pressure. Failure to upregulate paraventricular nucleus (PVN) Gαi_2_ proteins in response to elevated dietary sodium intake causes renal nerve-dependent sympathoexcitation, renal sodium retention and neuroinflammation resulting in the development of the salt sensitivity of blood pressure. Image was generated using BioRender.

To confirm a direct role of PVN-specific Gαi_2_ proteins in the observed development of salt sensitivity we conducted studies in which PVN specific Gαi_2_ proteins were down-regulated by bilateral PVN infusion (confirmed pharmacologically and histologically). These studies confirmed that PVN-specific Gαi_2_ downregulation results in the development of renal nerve-dependent salt sensitivity of blood pressure and renal sympathoexcitation (38). Extending our insight into the potential mechanisms driving the observed increase in renal sodium retention following Gαi_2_ protein downregulation we observed increased renal nerve-dependent activity of the sodium chloride cotransporter (NCC) (38). The NCC is critical to the fine tuning of sodium reabsorption and it has recently been demonstrated that increased sympathetic outflow can drive the expression and activity of the NCC to result in salt sensitive hypertension (9–12). Collectively, these data demonstrate the conserved role of brain Gαi_2_ proteins as a sodium-activated “anti-hypertensive” central pathway which regulates renal nerve-mediated sympathoinhibitory and natriuretic responses to prevent the development of salt sensitive hypertension (**Figure 2** and **Figure 3**).

**Figure 4:**
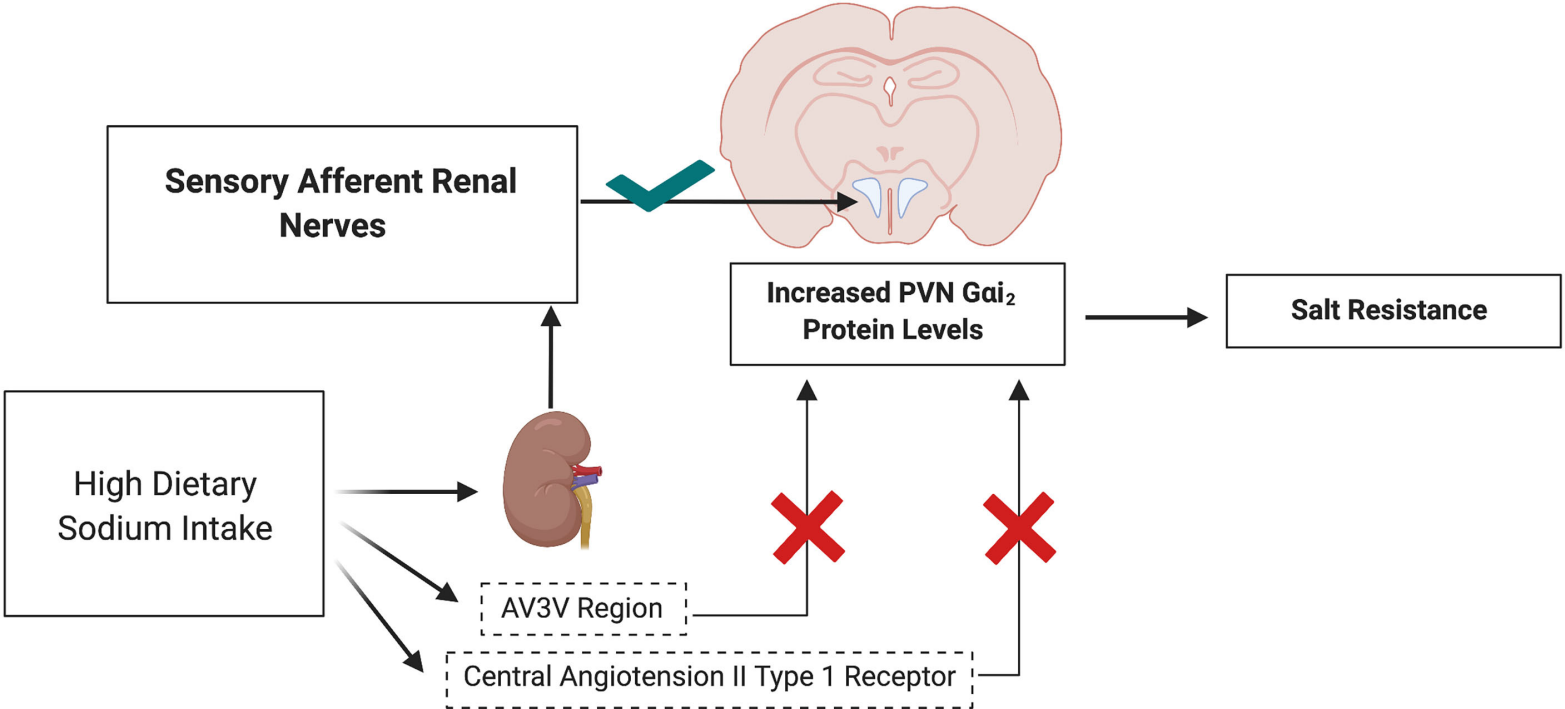
Schematic representation of upregulation of PVN Gαi_2_ protein-dependent responses to elevated dietary sodium intake in salt resistance. Upregulation of PVN specific Gαi_2_ is mediated by the peripheral sensory afferent renal nerves and does not depend on the AV3V sodium sensitive region or actions of central angiotensin II type 1 receptors. Image was generated using BioRender.

To investigate the potential mechanisms by which PVN Gαi_2_ proteins are endogenously up regulated by high dietary salt intake we investigated the contribution of the sodium sensitive peripheral sensory afferent renal nerves (46) and the anteroventral third ventricle (AV3V) region. In these studies, we observed that dietary salt evoked up regulation of PVN Gαi_2_ proteins occurs independently from the AV3V region but is dependent on the presence of the peripheral sensory afferent renal sympathetic nerves (40). Additionally, pharmacological blockade of central angiotensin II type 1 receptors does not attenuate the salt sensitivity of blood pressure in rats in which central Gαi_2_ proteins are down regulated - indicating that the development of Gαi_2_ protein dependent salt sensitive hypertension occurs independently of the actions of the brain angiotensin II type 1 receptor (40) (**Figure 4**).

## PVN Gαi_2_ Proteins and Neuroinflammation

PVN-specific upregulation of pro-inflammatory cytokines has been demonstrated in several animal models of hypertension including salt sensitive hypertension. In the DSS rat a high salt diet evokes PVN neuroinflammation (47) which may contribute to sympathoexcitation as neuroinflammation as multiple cytokines have been shown to affect the excitability of neurons (48–50). To assess the potential impact of Gαi_2_ proteins on neuroinflammation we assessed inflammation in the PVN and subfornical organ (SFO) in the presence and absence of Gαi_2_ proteins dietary a high salt intake. Our control studies in animals maintained on a normal salt intake reveal that central Gαi_2_ protein downregulation does not evoke neuroinflammation during standard salt intake – a setting when animals remain normotensive and in sodium balance (51). However, during high salt intake central ODN-mediated Gαi_2_ protein downregulation evoked sympathoexcitation, salt sensitive hypertension and PVN, but not SFO, microglial activation and production of the pro-inflammatory cytokines IL-1β, IL-6 and TNFα (51). In this setting we have established microglia as the potential source of PVN neuroinflammation and cytokine production as minocycline-mediated suppression of microglial activation attenuated PVN cytokine production and the magnitude of salt sensitive hypertension (51). Collectively, these data suggest a role of central Gαi_2_ proteins in maintaining an anti-inflammatory environment in the PVN in salt resistant rats during elevated dietary sodium intake. At present it remains unknown if central Gαi_2_ proteins influence systemic inflammatory processes or inflammation in other organ systems (e.g., the kidney or vasculature).

## Neuro-anatomical Localization of PVN Gαi_2_ Expressing Neurons

**Figure 5:**
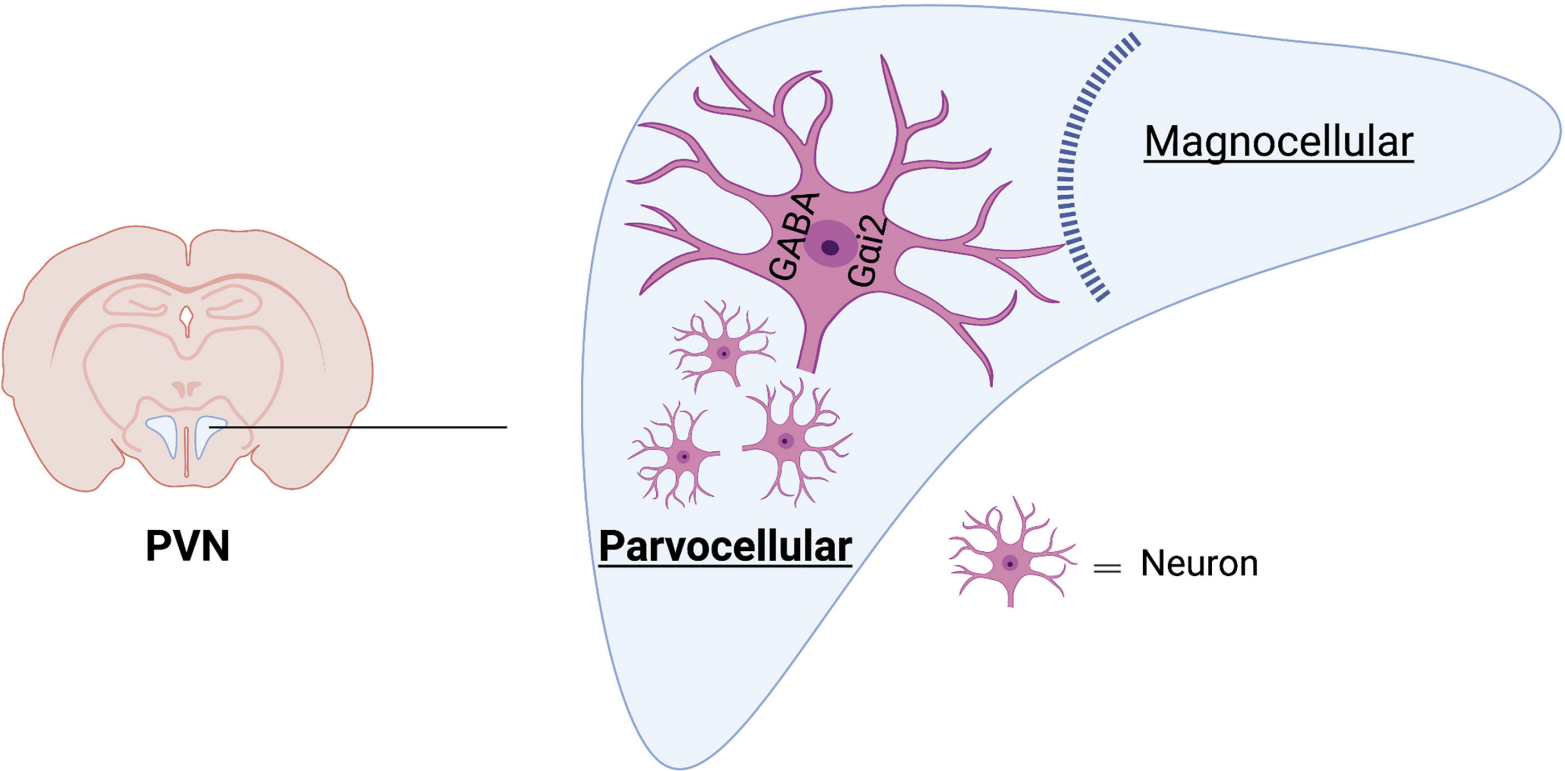
Schematic representation of neuroanatomical localization of Gαi2 mRNA-expressing neurons in PVN. Gαi2 mRNA expressing neurons are highly localized within the parvocellular region of the PVN and strongly colocalize with GABAergic neurons. Image was generated using BioRender.

Despite our extensive work localizing the effects of central Gαi_2_ proteins on sodium excretion and blood pressure regulation to the PVN, immunoblotting of PVN tissue punches does not provide insight into the neuroanatomical location of PVN Gαi_2_ positive neurons. It is well established the PVN coordinates neural and hormonal responses to alterations in plasma sodium and osmolality by two distinct cell types – the parvocellular and magnocellular neurons, respectively. The parvocellular division of the PVN is comprised of sympathetic regulatory neurons and neuroendocrine neurons (52) and the magnocellular division has oxytocin and vasopressin containing neurons (53). To conduct the neuroanatomical characterization of PVN Gαi_2_ expressing neurons we performed *in situ* hybridization on the PVN from male and female Sprague Dawley rats (54). Gnai2 mRNA, used as a marker of Gαi_2_ expressing neurons, was highly localized within the parvocellular region of the PVN and localization was similar between male and female animals. Gnai2 mRNA colocalized with 85% of GABA-expressing, 75% of corticotropin-releasing hormone and 28% of glutamatergic neurons at the level 2 of the PVN. Additionally, Gnai2 neurons exhibited a lower degree of colocalization with tyrosine hydroxylase (33%), oxytocin (6%) and arginine vasopressin expressing (10%) neurons in the PVN (54). Based on our data, Gαi_2_ expressing neurons are predominantly located in the sympathetic parvocellular division of the PVN with minimal expression in the magnocellular region of the PVN (**Figure 5**). The strong colocalization of Gnai2 with GABAergic neurons suggests a potential role of GABA in Gαi_2_ protein-mediated sympathoinhibition versus an influence on oxytocin and vasopressin positive neurons.

## Impact of Central Gαi_2_ Proteins on Acute and Chronic Neuronal Activation

To examine the impact of selective targeted downregulation of central Gαi_2_ proteins on acute sodium-evoked PVN neuronal activation we assessed alterations in PVN c-Fos immunoreactivity as a marker of neuronal activation in response to an acute 3M NaCl load. Hypertonic saline-induced c-Fos staining was significantly attenuated in the medial parvocellular (MP), ventrolateral parvocellular (VLP) and lateral parvocellular (LP) sub-nuclei of PVN in Gαi_2_ ODN pre-treated rats compared to control SCR ODN pre-treated rats with intact Gαi_2_ proteins (42). Additionally, we observed a significant suppression of circulating levels of plasma norepinephrine levels at 10 minutes post-3M NaCl infusion was observed in SCR ODN pretreated rats that was abolished in Gαi_2_ ODN pre-treated rats. These data suggest that central Gαi_2_ proteins are required to activate PVN parvocellular sympathoinhibitory neurons to suppress the release of norepinephrine in response to an acute 3M NaCl bolus. In contrast, the immunoreactivity of c-Fos positive neurons in the magnocellular division of the PVN was comparable between SCR and Gαi_2_ ODN pretreated animals in response to a 3M NaCl bolus Gαi_2_ proteins (42) and the 3M NaCl evoked increase in plasma AVP levels was identical in SCR and Gαi_2_ ODN pretreated groups. These findings are consistent with the results from *in situ* hybridization showing high parvocellular location of Gnai2 expressing neurons and minimal expression of Gnai2 in magnocellular vasopressin and oxytocin positive neurons (54). These data suggest that in response to an acute sodium challenge central Gαi_2_ protein downregulation impairs neuronal activation of sympathoinhibitory parvocellular, but not the neuroendocrine magnocellular division of the PVN.

Extending these findings from the acute to chronic setting we have reported that high dietary sodium intake in salt resistant Sprague Dawley rats receiving a central control SCR ODN infusion resulted in significant activation of PVN parvocellular neurons as assessed by FosB staining (marker of chronic neuronal activation of PVN). These animals, which up-regulate PVN specific Gαi_2_ proteins, remained normotensive and in sodium balance and exhibited suppression of global and renal sympathetic outflow at the same time point increased parvocellular FosB staining was detected (38). In contrast, central down-regulation of Gαi_2_ proteins during high dietary salt intake markedly attenuated PVN parvocellular FosB staining - indicating decreased neuronal activation. Accompanying decreased PVN neuronal activation we observed significantly elevated renal norepinephrine content and turnover, suggesting PVN Gαi_2_-dependent signaling regulates sympathetic outflow to the kidneys to influence renal sodium excretion and the salt sensitivity of blood pressure (38). Collectively, our data suggest that in response to acute and chronic sodium challenges central Gαi_2_ protein-mediated pathways are required to activate sympathetic-regulatory parvocellular neurons, but not neuroendocrine magnocellular neurons, to mediate sympathoinhibition, natriuresis and normotension.

## Gαi Proteins in Cardiovascular Tissues

Beyond our studies on the role(s) of central Gα-subunit proteins in blood pressure regulation it has been reported that differences in peripheral Gαi protein expression, in vascular and cardiac tissues, can also influence blood pressure in animal models. Increases in vascular expression of Gαi subunit proteins, which will reduce intracellular cAMP levels, have been reported in the Spontaneously Hypertensive Rat (SHR) (55), L-NAME hypertensive rat (56) and deoxycorticosterone acetate salt (DOCA-salt) (18) rat models of hypertension. In these models it is hypothesized that increased vascular levels of Gαi subunit proteins contributes to arterial stiffening and increased vascular tone evoking increases in blood pressure.

Further, alterations in Gαi protein expression and adenylyl cyclase activity have been reported in platelets (57) in human subjects with hypertension. In contrast to our data that central angiotensin II type 1 receptors play no role in Gαi_2_ protein dependent salt sensitivity of blood pressure (40) angiotensin receptor antagonism is able to attenuate the upregulation of Gαi proteins in vascular tissue and evoke reductions in blood pressure in the L-NAME hypertensive rat (56). Collectively, in concert with our published findings, these data suggest that there is a tissue-dependent role of Gαi proteins in the regulation of blood pressure.

## G Protein Subunit Alpha I2 Polymorphic Variance and Blood Pressure

Our experimental animal studies, described above, highlight the central role of the PVN Gαi_2_ proteins in the maintenance of salt resistance *via* renal nerve-dependent sympathoinhibitory mechanisms. However, expanding these findings by translational studies is essential for the development of therapeutic targets and screening approaches to identify salt sensitive subjects. Suggesting that GNAI2 polymorphic variance may be a potential biomarker for hypertension risk The Japanese Millennium Genome Project, which identified multiple single nucleotide polymorphisms (SNPs) that correlated with high blood pressure in Japanese individuals, identified a positive association with GNAI2 SNPs and hypertension (58). Additionally, studies in an Italian European cohort have suggested that a single C>G mutation in the GNAI2 promoter region reduces the binding of the specificity protein 1 (Sp1) transcription factor and is associated with hypertension (59).

Our recent studies have identified two additional SNPs in the human GNAI2 gene, rs2298952 and rs4547694, which significantly correlate with essential hypertension in UK BioBank data set of individuals of European ancestry (38). SNP rs4547694 has a Minor Allele Frequency (MAF) of 38.1%, suggesting that this GNAI2 SNP may be a prevalent marker that is associated with essential hypertension in at-risk individuals of European ancestry. To examine potential associations between GNAI2 polymorphisms and the salt sensitivity of blood pressure, as suggested by our animal studies, we examined the Genetic Epidemiology of Salt Sensitivity (GenSalt) data set in which the salt sensitivity of blood pressure was rigorously assessed. In an examination of 968 Chinese individuals in GenSalt we found a positive association between the GNAI2 SNP rs10510755 and the salt sensitivity of blood pressure (60). SNP rs10510755 was present in 118 of 369 (~32%) salt sensitive individuals in the GenSalt data set. These data, which require extensive validation in other data sets, suggest that GNAI2 polymorphic variance may represent a potential biomarker of the salt sensitivity of blood pressure in a sub-set of individuals to aid in cardiovascular risk stratification and the development of a rapid reliable method to assess the salt sensitivity of blood pressure.

## Summary and Perspectives

Hypertension and the salt sensitivity of blood pressure are critical public health issues. However, the mechanisms underlying the development and maintenance of salt sensitive hypertension are poorly understood. Our laboratory has advanced the understanding of the functional selectivity of central Gα-subunit proteins in the regulation of cardiovascular versus renal excretory function. We have revealed that central Gαz/q proteins modulate the release of vasopressin to influence aquaretic responses to pharmacological and physiological challenges. Our laboratory has reported that central Gαi_2_ proteins mediate the salt resistant, sympathoinhibitory, natriuretic and central anti-inflammatory responses to elevated dietary sodium intake and are essential to maintain natriuresis and sodium homeostasis in response to acute challenges to fluid and electrolyte balance. Further, we have shown that PVN specific Gαi_2_ proteins are critical to the maintenance of salt resistance and prevent the development of salt sensitive hypertension in rat models. Collectively, PVN Gαi_2_ signal transduction pathways have emerged as a novel therapeutic for the management of hypertension. Our human genetic polymorphism studies have shown a strong positive correlation between GNAI2 polymorphic variance and the salt sensitivity of blood pressure and hypertension in populations of different ancestry. These findings suggest that GNAI2 SNPs are a potential biomarker for the salt sensitivity of blood pressure that may aid in cardiovascular risk stratification and targeted reduction in dietary salt intake.

## Author Contributions

RA, JM, and RW conceived and designed research; RA and JM prepared figures; RA, and JM drafted manuscript; RA, JM, and RW edited and revised manuscript; RA, JM, and RW approved final version of manuscript.

## Funding

These studies were supported by National Heart, Lung, and Blood Institute (NHLBI) R01HL141406, R01HL139867, and National Institute on Aging (NIA) R01AG062515 and R01AG075963 to RW.

## Conflict of Interest

The authors declare that the research was conducted in the absence of any commercial or financial relationships that could be construed as a potential conflict of interest.

## Publisher’s Note

All claims expressed in this article are solely those of the authors and do not necessarily represent those of their affiliated organizations, or those of the publisher, the editors and the reviewers. Any product that may be evaluated in this article, or claim that may be made by its manufacturer, is not guaranteed or endorsed by the publisher.
